# Pulmonary Tuberculosis

**Published:** 1894-04-28

**Authors:** 


### Medical Progress.

PULMONARY TUBERCULOSIS *(continued)*.

*Chlorine* gas injected into pulmonary cavities is advocated by Shurly. After referring to the difficulty of localising and determining the size of these cavities, Shurly[1] discusses their treatment by the insertion of rubber tube and injection of chlorine gas. There are two chief dangers in incising these cavities—(1) hæmorrhage, and (2) infection of the freshly incised surfaces. Chlorine is capable of destroying the virulence of tubercle and caseous material. The diluted chlorine was obtained by pumping air with a common rubber bulb through a Wolff's bottle, previously filled with gas, and connected with the drainage tube which had been inserted in the cavity. Twelve to fifteen bulbsful may be thus introduced, and this may be repeated every two to four hours. There was very little uneasiness, and little or no cough after it; in fact, it exercised a soothing influence. The author refers to a case successfully treated in this way, which he describes as croupous pneumonia with empyema. Tubercle bacilli were present in the sputum, and a communication existed between the lung and the pleural cavity. He afterwards relates in detail two cases of advanced phthisis also treated with chlorine in this way. Both died. He draws attention to the following points: (1) The cavity should be opened near the apex without resecting ribs and by using the galvano-cautery in cutting through the lung, and (2) an antiseptic gas should be used instead of a fluid. If the layers of the pleura are not adherent it is inadvisable to proceed further.

*Cinnamic acid*[2] has been extensively used by Professor Landerer, of Leipzig. Dr. Paul Richter has conducted some experiments on rabbits which go far towards convincing us that Landerer's conclusions are well founded. Richter inoculated ten rabbits with fresh pure culture of tubercle bacilli. By the nineteenth day three rabbits had died, the last two with definite tubercular lesions, and thus it was assumed that the remaining seven were tuberculous.

One was reserved as a control animal, the other six were injected with Landerer's 5 per cent. solution of cinnamic acid two or three times a week for six months. Two animals died spontaneously, and the control animal died in seven months. The others were killed in about six months, and on examination of the tissues showed, first, an inflammatory area surrounding each tubercle, chiefly associated with capillary dilatation, extravasation of serum, and leucocytosis. Later the nodule had become surrounded by a wall of leucocytes, and at the same time multinuclear leucocytes had begun to wander into the tubercle. Finally the tubercle was surrounded by a connective tissue capsule, and by that time bacilli had almost entirely disappeared.

Landerer's results are embodied in a small work issued by him. He used an emulsion as follows:

Cinnamic acid, powdered, 5 grains; oil of almonds, 10 grains; the yolk of an egg; solution of sodium chloride, 0·7 per cent. to 100 grains.

The acid is first well ground in a mortar, a little almond oil added, and the trituration continued. The rest of the oil is then rubbed in and the yolk added. The solution of salt is then added drop by drop. The mixture after standing a few days should be perfectly homogeneous, and should deposit no crystals of cinnamic acid.

For alkalising the emulsion a 7·5 per cent. solution of sodium hydrate is used, about five drops of this are added to the cubic centimetre of emulsion. The emulsion should be made, and no crystals of the acid should be detected by the microscope.

The emulsion is injected directly into the cephalic vein in the arm, it being made to stand out prominently as though it was intended to bleed. At first 0·1 ccm. is the dose, of course injected with a perfectly sterile syringe. The dose is gradually increased to 0·25, or even to 0·4 ccm.

No symptoms beyond perhaps a slight transient local burning sensation should follow the injection; depression is a sign of too large a dose. As regards frequency, Landerer gives two injections weekly as a rule, but the best results are obtained when 0·1 or 0·2 ccm. are given every second day. The treatment should be continued for three months at least, or at any rate for a month after bacilli have disappeared from the sputum in pulmonary tuberculosis.

Laryngeal tuberculosis he treated by painting the parts with cinnamic acid and alcohol 1·20, or better, the acid with glycerine, 1·20 or 1·10 in conjunction with intravenous injections.

He claims to have succeeded in improving 66⅔ per cent. of cases of pulmonary excavation when there was no considerable degree of fever. While cases of chronic tuberculosis without pulmonary cavities give an almost absolutely good prognosis.

For further details, especially in reference to surgical tubercular disease, the reader is referred to the original work of Landerer, and to the valuable review of the recent literature of the cinnamic acid treatment by J. C. Warbasse, "Annals of Surgery," (loc. cit.), from which our notes are taken.

*Guaiacol.—As an antipyretic.* Is guaiacol absorbed by the skin? Guaiacol has recently been used as an antipyretic in many febrile conditions, but our remarks will refer chiefly to its use in the pyrexia of phthisis. J. M. Da Costa, and Robilliard[3] have found from clinical experience that the local application of guaiacol diminishes the temperature even more rapidly and constantly than the sulphate of quinine. The drug is applied over the chest, or arms, or thighs, and half a gramme is generally sufficient.

Stolzenburg[4] has tried in Senator's clinic the external use of guaiacol as an antipyretic in the way recommended by Sciolla. Most were instances of the pyrexia of phthisis, and only two or three of acute disease. Two ccm. were found to be sufficient for the purpose, and in feeble patients one ccm. should be used at first. In the course of the next few hours the temperature fell, with copious sweating, but it soon rose again, often with shivering, attaining a greater height than before. In most cases the treatment had to be discontinued, owing to objection on the part of the patients. The smell is also unpleasant. No real influence on the disease could be made out. Its continued use cannot be recommended. Larger doses may produce collapse. The absorption through the skin is beyond question.

That absorption does take place by skin has been demonstrated by Linnossier and Lannois,[5] for the effect was just as marked in their experience when the patient breathed through a tube opening outside the room. On the other hand, M. Guinard[6] found that the fall of temperature following the application of guaiacol was not the result of absorption. He showed (*a*) that it is not absorbed unless it is allowed to enter by the respiratory passages; (*b*) that the quantity of vapour that can be absorbed in this way is not sufficient of itself to produce the fall of temperature, and from experiments upon animals, that it acts upon the centres of thermogenesis by exciting the peripheral nerve terminations, and then reflexly upon the functions of the centres.

Cohen[7] employed guaiacol locally applied in two cases of phthisis.

*Salol.*—Dr. A. Lutz[8], a Brazilian physician, speaks of the excellent effects obtained by himself in the treatment of pulmonary tuberculosis by the administration of salol in doses of from one to two drachms in the twenty-four hours (the ordinary dose being a drachm and a half in the twenty-four hours). Salol differs from the other remedies employed in cases of phthisis (creasote, guaiacol, &c.), which are most active during the apyretic stages of the disease inasmuch as it is especially beneficial in the febrile cases, *i.e.*, in the acute or sub-acute forms of pulmonary tuberculosis. Under such circumstances the administration of salol is followed at the end of a week or a fortnight by a gradual diminution of the fever, night sweats, cough, and expectoration. The treatment is usually well borne, except in cases of kidney complications, when salol is evidently contra-indicated. Should the patient complain of nausea, noises in the ears, or vertigo under the influence of salol, all these manifestations rapidly disappear on suspending the administration for some time, after which the treatment may be resumed and continued without further trouble. Dr. Lutz believes that the favourable effects produced by salol in the cases of phthisis with a high temperature under his observation are due to the action of the remedy, not so much on the bacilli of tuberculosis, as on the other bacteria which coexist with the latter and contribute to the general infection of the organism, as well as to the local destruction of the pulmonary tissue. According to the author, the mixed pulmonary tubercular infection becomes transformed under the influence of salol into a purely tubercular infection. As a result of this transformation the fever subsides, while the progress of the affection and the destruction of the pulmonary tissue is interfered with.

Tuberculin has been given by Schiess Bey and Kartulis[9] in forty-eight tuberculous patients. Their results were favourable, but they considered that the climate of Egypt conduced to the good result, it being, in their opinion, especially suitable for the tuberculin treatment.

Experimental inoculation for diagnosis has been utilised by Dr. T. Harris[10] in regard to one obscure case under his care. The doubtful points in the physical signs were not cleared up by the examination of the sputum. Dr. Harris could not be sure whether tubercle bacilli were present for reasons stated. Professor Sheridan Delépine was able to prove that the sputum contained the virus of tubercle and thus, although the patient on a second visit presented no definite physical indications of the nature of the mischief, Dr. Harris was able to be certain as to the real character of the affection, and to treat accordingly.

Dr. Klein has for a considerable time been conducting experiments to determine the action of the products of the growth of the bacillus pyocyaneous as a possible therapeutic agent in tuberculosis. His experiments seem to prove that the toxine of this microbe has an inhibitory action on the growth of the bacillus of tubercle, or at least is able to modify and exhibit the tubercular process. At present the results are only encouraging, and are not sufficiently conclusive to warrant the process being utilised clinically.

[1] Journ. Amer. Med. Ass., Aug. 26, 1894, and Brit. Med. Journ., Jan. 20, 1894. [2] Annals of Surgery, Jan., 1894. [3] Gaz. Med. de Paris. [4] Berl. Klin Woch., Jan. 29, and Brit. Med. Journ., Feb. 24, 1894. [5] La Sem. Med., Feb. 7, 1894. [6] Bull. Gen. de Thera., p. 339, 1893. [7] Phil. Med, New., Sept. 30. [8] Fort. de Med., No. 23, Dec., 1893, and Med. Week, Dec. 15, 1893. [9] Zeit. f. Hygiene, No. 15, Part 2. [10] Lancet, Dec. 9, 1893.

